# Rapid, simple, and sensitive detection of the *ompB* gene of spotted fever group rickettsiae by loop-mediated isothermal amplification

**DOI:** 10.1186/1471-2334-12-254

**Published:** 2012-10-11

**Authors:** Lei Pan, Lijuan Zhang, Guiqiang Wang, Qinghui Liu

**Affiliations:** 1 Department of Rickettsiology, National Institute of Communicable Disease Control and Prevention, China CDC, Changping P.O.BOX5, Beijing, 102206, People’s Republic of China; 2 Department of Infectious Diseases, Peking University First Hospital, Xishenku Street 8, Xicheng District, Beijing, 100034, People’s Republic of China; 3 Laizhou People’s Hospital, Wenhua East Road 288, Laizhou, Shandong Province, 261400, People’s Republic of China

**Keywords:** Rickettsia, LAMP assay, Molecular detection, Spotted fever, Clinical microbiology

## Abstract

**Background:**

Spotted fever caused spotted fever group rickettsiae (SFGR) is prevalent throughout China. In this study, we describe a rapid, simple, and sensitive loop-mediated isothermal amplification (LAMP) assay targeting the *ompB* gene of spotted fever group rickettsiae ideal for application in China. The LAMP assay has the potential to detect spotted fever group rickettsiae early in infection and could therefore serve as an alternative to existing methods.

**Methods:**

A set of universal primers which are specific 7 common species of spotted fever group rickettsiae in China were designed using PrimerExplorer V4 software based on conserved sequences of *ompB* gene. The sensitivity, specificity and reproducibility of the LAMP were evaluated. The LAMP assay for detecting SFGR was compared with conventional PCR assays for sensitivity and specificity in early phase blood samples obtained from 11 infected human subjects.

**Results:**

The sensitivity of the LAMP assay was five copies per reaction (25 μL total volume), and the assay did not detect false-positive amplification across 42 strains of 27 members of the order *Rickettsiales* and 17 common clinical pathogens. The LAMP assay was negative to typhus group rickettsiae including *R. prowazekii* and *R. typhi* for no available conserved sequences of *ompB* was obtained for designing primers.

To evaluate the clinical applicability of the LAMP assay, a total of 11 clinical samples, 10 samples confirmed serologically (3 cases), ecologically (1 case), by real-time polymerase chain reaction (PCR; 2 cases), ecologically and by real-time PCR (1 case), and serologically and by real-time PCR (3 cases) were analyzed by the *ompB* LAMP assay. Data were validated using a previously established nested PCR protocol and real-time PCR. A positive LAMP result was obtained for 8 of the 10 confirmed cases (sensitivity, 73%; specificity, 100%), while none of these samples were positive by nested PCR (sensitivity, 0%; specificity, 100%).

**Conclusions:**

The LAMP assay described here is the most reliable among the three methods tested and would be an ideal choice for development as a rapid and cost-effective means of detecting SFGR in China.

## Background

Spotted fever is caused by obligate (Gram-negative) intracellular spotted fever group rickettsiae (SFGR) [1]. SFGR are widely distributed throughout the world, causing both emerging and re-emerging infectious diseases [2,3]. People working in wild fields, including farm workers, forest workers, field soldiers, and travelers, are the main populations susceptible to SFGR infection. In 1984, only five species of SFGR were confirmed as human pathogens but that number has since risen to more than ten, such us *Rickettsia massiliae*, *R. raoultii, R. parkeri, R. africae, R. felis, R. japonica, R. peacokii, R. helvetica, R. montanensis, R. monacensis, R. rhipicephali*. There are three main pathogenic SFGR in China: *R. sibirica*[4], *R. heilongjiangensis*[5], and *R. sibirica mongolotimonae*[6].

Unrecognized pathogens, combined with the lack of a rapid and sensitive diagnostic method for identifying SFGR, may be responsible for causing delayed treatment or misdiagnosis that could lead to the development of severe disease and fatality [7]. The greatest challenge to clinicians is not therapy but the difficult diagnosis during the early phase of infection. Conventional serodiagnosis requires serum from both the acute and convalescent stages of infection, the latter of which would be unavailable at early diagnosis. Although SFGR can be isolated and reliably identified from samples cultivated in the laboratory, this approach for early diagnoses also has its drawbacks, as this method is generally associated with low isolation rates and requires sophisticated and expensive equipment. Other methods that have been applied for this purpose, such as those involving the polymerase chain reaction (PCR) (i.e., conventional, nested, and real-time PCR), also require expensive, specialized instruments those are not widely available.

Loop-mediated isothermal amplification (LAMP) is a novel nucleic acid amplification method that was developed in 2000 [8]. LAMP is a highly sensitive, specific and simple technique that under isothermal conditions (60-65°C) can generate up to a 10^9^-fold amplification in less than an hour, making LAMP a rapid and simple diagnostic tool for identifying SFGR. In this study, we aimed to develop a rapid, sensitive and specific assay targeting the *ompB* gene to detect SFGR infection in humans from rural areas of China.

## Methods

### Clinical samples

Between 2007 and 2009, we obtained 11 samples from clinical SFGR cases from four different hospitals (Peking University First Hospital, Laizhou People’s Hospital, Beijing Friendship Hospital, and 302 Military Hospital of China). This study was approved by the Human Research Ethics Committee of the Chinese Center for Disease Control and Prevention (China CDC). Before entry into the study, each patient was informed about the purpose and procedures of the research and written consent was obtained. Two milliliters of ethylenediaminetetraacetic acid (EDTA)-anticoagulated blood and 2 mL of non-anticoagulated blood were collected during the acute phase of illness for culture and for the detection of IgM and IgG antibodies, respectively. DNA was extracted from the remaining clots for nested PCR [9], real-time PCR [10], and the LAMP assay. A second serum sample was collected during the convalescent stage (2-4 weeks after acute phase) to detect IgG antibodies. Each case was confirmed by indirect fluorescent antibody analysis (IFA; four-fold increase or decrease in IgG antibody titer), as recommended by the World Health Organization (WHO), and the analysis of clinical criteria was able to exclude other febrile illnesses [7,10-12]. All the patients were eventually cured by administration of specific antibiotic therapy for rickettsiosis.

### Laboratory strains

The specificity of the LAMP assay was determined using a total of 42 strains of the order *Rickettsiales* (Table 1). These strains were provided by Dr Raoult D from the WHO Collaborating Centre for Rickettsial Reference and Research (Marseille, France). Other common clinical pathogenic bacteria were obtained from the relevant department of the China CDC (Table 1). Genomic DNA was extracted from cultured cells using the QIAamp DNA Mini Kit (Qiagen, Hilden, Germany). In addition, total blood DNA from healthy humans, cattle, horses, goats, and mice was isolated for use as negative controls. Before being used to determine the specificity of the LAMP assay, each bacterial DNA sample was first pre-screened by PCR using universal prokaryotic bacterial 16S rRNA primers, as previously described [13]. Genomic DNA from the 42 strains was tested by LAMP to determine the specificity of the *ompB* LAMP assay. All detection assays were performed in triplicate.

**Table 1:** Laboratory strains used for determining the specificity of the LAMP assay and aligned sequences for designing primers

**Members of the order Rickettsiales**	**The related species**	**Aligned sequences**
*Rickettsia prowazekii*	*Bartonella henselae*	*Astrakhan rickettsia* (AF123708.1)
*Rickettsia typhi*	*Bartonella quintana*	Israeli tick typhus rickettsia (AF123712.1)
*Orientia tsutsugamushi* (types Karp, Kato and Gilliam)		*Rickettsia mongolotimonae* (AF123715.1)
*Anaplasma phagocytophilium* strains Webster, MRK, Slovienie and MD	**Other common clinical pathogenic bacteria**	*Rickettsia parkeri* (AF123717.1)
*Rickettsia sibirica*	*Coxiella burnetii*	*Rickettsia* sp. S (AF123720.1)
*Rickettsia conorii*	*Borrelia burgdorferi (2)*^*a*^	*Rickettsia conorii* strain Seven (AF123721.1)
*Rickettsia honei strain marmionii*	*Escherichia coli (3)*^*a*^	*Rickettsia sibirica* (AF123722.1)
*Rickettsia akari*	*Vibrio cholerae*	*Rickettsia conorii* strain Indian tick typhus rickettsia (AF123726.1)
*Rickettsia rickettsii*	*Bacillus anthracis*	*Rickettsia heilongjiangensis* (AY260451.1)
*Rickettsia africa*	*Haemophilus influenzae (2)*^*a*^	*Rickettsia heilongjiangensis* variant extremiorientalis (AY280712.1)
*Rickettsia parkeri*	*Listeria spp.*	*Rickettsia* sp. BJ-90 outer (AY331393.1)
*Rickettsia japonica*	*Legionella spp.*	*Rickettsia mongolotimonae* isolate URRMTMFEe65 (DQ097083.1)
*Rickettsia slovaca*	*Yersinia pestis*	*Rickettsia rickettsii* strain Dv090589 (GU395293.1)
*Rickettsia aeschlimannii*	*Shigella dysenteriae (3)*^*a*^	*Rickettsia sibirica* strain RH05 (HM050273.1)
*Rickettsia montanensis*	*Neisseria meningitides*	*Rickettsia rickettsii* (X16353.1)
*Rickettsia helvetica*	*Leptospira spp.*	*Rickettsia japonica* (AB003681.1)
*Rickettsia felis*		*Rickettsia africae* (AF123706.1).
*Rickettsia australis*		
*Rickettsia canadensis*		
*Rickettsia bellii*		
*Rickettsia heilongjiangensis*		
*Ehrlichia chaffeensis*		

Note: ^a^: The number in brackets indicates the number of the isolates used in the study.

### LAMP primer design

**Figure 1:**
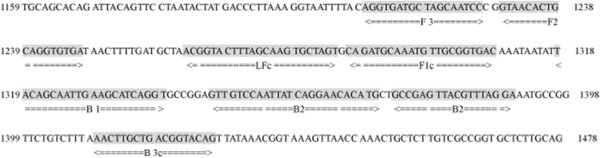
**The rickettsial *ompB *gene indicating names and binding sites of primers for LAMP.**

A set of universal primers complementary to the *ompB* of *Rickettsia* sp. BJ-90 (GenBank Accession: AY331393) was designed using PrimerExplorer V4 software (http://primerexplorer.jp; Eiken Chemical Co., Ltd., Tokyo, Japan) based on conserved sequences determined by the nucleotide alignment of 17 *ompB* GenBank entries (Table 1). These primers were assumed to be specific for SFGR since no available conserved sequences of the *ompB* gene were found in the typhus group of rickettsiae, such as *R. prowazekii* and *R. typhi*. The primers were synthesized by Sangon Biotech (Shanghai, China). The primer sequences and their positions relative to the *ompB* gene are shown in Table 2 and Figure 1.

**Table 2:** **Nucleotide primer sets used for the *ompB *LAMP assay**

**Primer type**	**Positions**	**Sequence 5**^**′**^**-3**^**′**^
FIP(F1c-F2)	1287-1308/1230-1247	GTCACCGCAACATTTGCATCTG-GTAACACTGCAGGTGTGAT
BIP(B1c-B2)	318-1339/1373-1390	TACAGCAATTGAAGCATCAGGT-TCCTAAACGTAACTCGGC
F3	1210-1227	AGGTGATGCTAIIAATCC
B3	1410-1427	CTGTACCITCAGCAAGTT
LF	1263-1285	ACTAGCACTTGCTAAAGTACCGT
LB	1346-1370	GTTGTCCAATTATCAGGAACACATG

### Reference plasmid

To determine the sensitivity of the LAMP assay, a recombinant plasmid containing the target sequence of the *Rickettsia* sp. BJ-90 (GenBank Accession: AY331393) *ompB* gene was constructed. The sequence located between the F3 and B3 primer binding sites was amplified using primers OP1 (5^′^-CACCGCCAGCGTTCCCTAT-3^′^) and OP2 (5^′^-AACTGCTGAAATGGCTGGAC-3^′^). The resulting PCR products (624 bp) were cloned into the pEASY-T1 vector using the pEASY-T1 Cloning Kit (TransGen, China). The recombinant plasmid was quantified by NanoPhotometer (Implen, Germany) and serially diluted (to concentrations of 5^5^, 5^4^, 5^3^, 5^2^, 5^1^, and 5^0^ copies/μL) to evaluate the limit of detection and reproducibility of the *ompB* LAMP assay.

### LAMP reaction

All LAMP reactions were performed using the Loopamp Kit (Eiken Chemical Co., Ltd., Tokyo, Japan) in a 25 μL reaction volume containing 1.6 μM of each of the FIP and BIP primers, 0.8 μM of the LF and LB primers, 0.2 μM of the F3 and B3 primers, 20 mM Tris–HCl (pH 8.8), 10 mM KCl, 8 mM MgSO_4_, 10 mM (NH4)_2_SO_4_, 0.1% Tween-20, 0.8 M betaine, 1.4 mM of each deoxynucleoside triphosphate (dNTP), and 1 μL *Bst* DNA polymerase (8 U/μL). The reaction was incubated in a real-time turbidimeter (model LA200, Teramecs, Tokyo, Japan) at 63°C for 60 min and then at 80°C for 5 min to terminate the reaction. Positive and negative samples were distinguished by a turbidity cutoff value of 0.1. After amplification, the LAMP products were examined by electrophoresis on 2% agarose gels stained with ethidium bromide or by visual inspection after the addition of 1 μL of 1000× concentrated SYBR Green I.

### Evaluation of the sensitivity, specificity, and reproducibility of the LAMP assay

To compare the sensitivities of the *ompB* LAMP assay and general PCR, the serially-diluted reference plasmid (at concentrations of 5^5^, 5^4^, 5^3^, 5^2^, 5^1^, and 5^0^ copies/μL) containing the target DNA was used to first define the limit of detection. A general PCR using primers OP1 and OP2 was performed as a 25 μL reaction containing 0.2 μM of each primer, 0.4 mM of each dNTP, and 1 U of *Taq* DNA polymerase. PCR was performed 35 times using a denaturation step of 94°C for 50 s followed by annealing at 52.5°C for 30 s and extension at 72°C for 45 s. The PCR products were electrophoresed on a 1% TBE agarose gel and stained with ethidium bromide (1 μg/mL).

To evaluate the reproducibility of the LAMP assay, the serially-diluted reference plasmids were amplified in two ways: five different times on the same day or once on each of five consecutive days. The intra-assay coefficient of variation (CVi) and inter-assay coefficient of variation (CVo) were analyzed at the time of peak precipitation, as determined using a real-time turbidimeter, as previously described [14,15]. Statistical analysis was conducted using SAS software (version 9.1; SAS Institute, Cary, NC).

## Results

### Specificity of LAMP

To determine the specificity of the LAMP assay for the detection of SFGR, 27 members of the order *Rickettsiales* were tested for amplification that included seven species of the spotted fever group (*R. sibirica*, *R. conorii*, *R*. *rickettsii*, *R. africa*, *R. parkeri*, *R. japonicai*, and *R. heilongjiangensis*). All seven SFGR tested by this method were positive and other common pathogens were not detected by LAMP. These results indicate that the *ompB* LAMP assay was specific for SFGR. The LAMP assay developed here did not detect *R. prowazekii* and *R. typhi* even though both possess an *ompB* gene but no conserved sequences where primers were design are present in these bacteria.

### Sensitivity of LAMP

**Figure 2:**
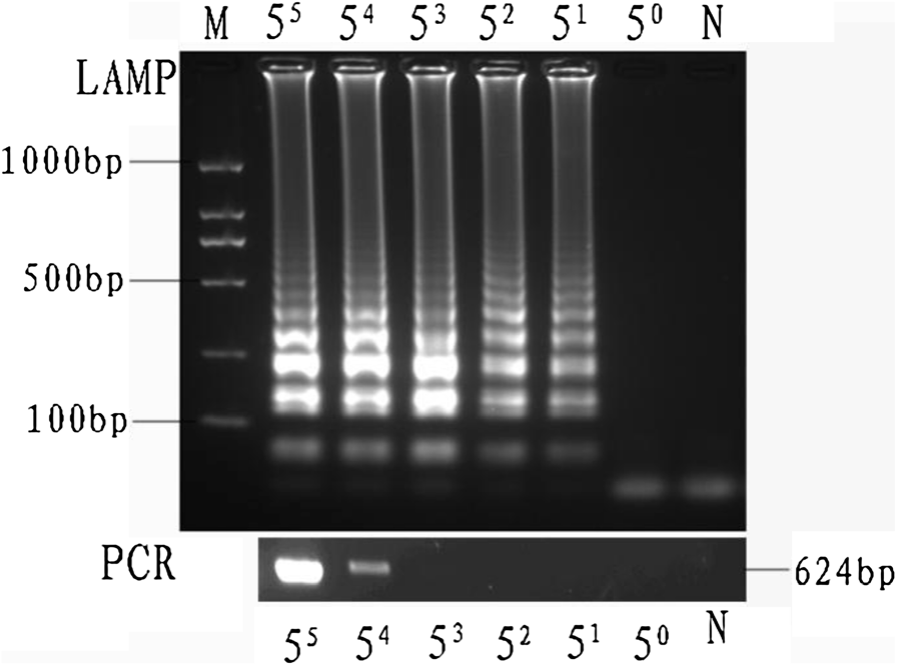
**Comparison of the detection limit of LAMP and general PCR, as observed after agarose gel electrophoresis.** Lane M, DNA marker; lanes 5^5^, 5^4^, 5^3^, 5^2^, 5^1^, and 5^0^ indicate the number of gene copies/μL; lane N, negative control.

**Figure 3:**
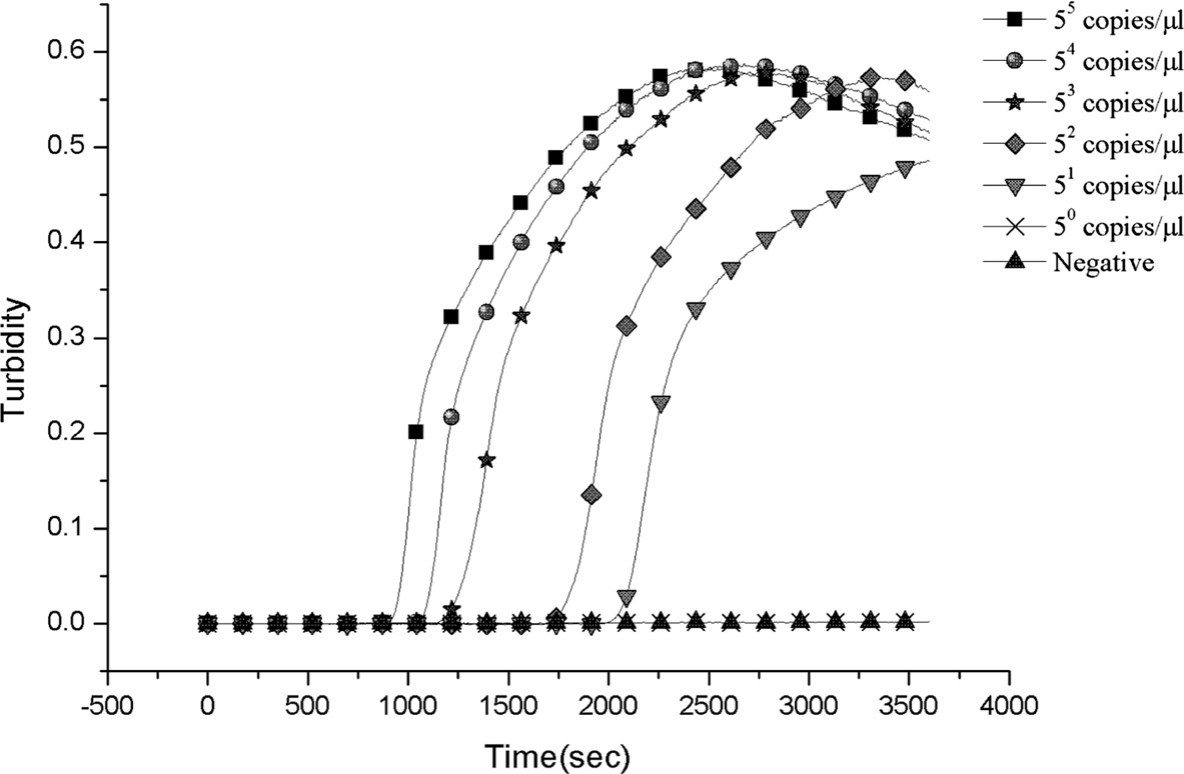
Sensitivity of the LAMP assay as monitored by the real-time measurement of turbidity.

The limits of detection of LAMP and PCR for the *ompB* gene were 5 and 625 copies per reaction, respectively (Figures 2 and 3). Thus, the LAMP assay is 125-fold sensitive than conventional PCR for detecting SFGR.

### Reproducibility of LAMP

The minimal detection levels, CVi and CVo, of the LAMP assay were 1.58 and 3.11%, respectively (Table 3).

**Table 3:** Summary of the CVi and CVo of the time of peak precipitation of serially-diluted plasmids

**Concentration copies/μL**	**CVi%**^**a**^	**CVo%**^**b**^
5^5^	1.20%	2.22%
5^4^	1.40%	2.53%
5^3^	1.55%	2.81%
5^2^	1.58%	3.11%

^a^Intra-assay coefficient of variation.

^b^Inter-assay coefficient of variation.

### Examination of clinical samples

Of the 11 samples that were assayed, seven cases of SFGR infection were confirmed in our laboratory. While one of the SFGR assignments was made by analysis of bacterial cultures individually, the remaining three were confirmed individually by IFA of acute- and convalescent-phase serum samples, two were confirmed individually by real-time PCR, one was confirmed by both analysis of bacterial cultures and real-time PCR, and three were confirmed by both IFA of acute- and convalescent- phase serum samples and real-time PCR. To evaluate the utility of LAMP for use with clinical samples, the 11 samples analyzed by IFA were also subjected to analysis by LAMP and nested PCR (Table 4). Eight of the 10 confirmed cases of SFGR were positive by the LAMP assay. In contrast, nested PCR did not yield any positive identification.

**Table 4:** Comparison of the LAMP assay with nested PCR assay for the detection of the spotted fever rickettsiae group in clinical samples^a^

**No. of cases**	**Diagnosis type**	**LAMP**	**Nested PCR**	**Real-time PCR**
1	*gltA* gene amplified	Pos.	Neg.	Pos.
2	*gltA* gene amplified	Pos.	Neg.	Pos.
3	4-fold IgG titer increase	Pos.	Neg.	Neg.
4	Culture confirmed and *gltA* gene amplified	Pos.	Neg.	Pos.
5	4-fold IgG titer increase	Pos.	Neg.	Neg.
6	4-fold IgG titer increase and *gltA* gene amplified	Pos.	Neg.	Pos.
7	4-fold IgG titer increase and *gltA* gene amplified	Pos.	Neg.	Pos.
8	Suspected case	Neg.	Neg.	Pos.
9	Culture confirmed	Neg.	Neg.	Neg.
10	4-fold IgG titer increase	Neg.	Neg.	Neg.
11	4-fold IgG titer increase and *gltA* gene amplified	Pos.	Neg.	Pos.

^a^ All assays were performed in triplicate.

## Discussion

Although SFGR have been monitored in China for nearly 30 years, infection with SFGR remains widely undiagnosed, a problem that could be the cause of delays in treatment or misdiagnoses that could lead to severe disease and fatal outcomes [3]. The WHO Collaborating Centre for Rickettsial Reference and Research (Marseille, France) regards the IFA as the “gold standard” for the serological testing of rickettsial diseases. The drawback of this method, however, is that it requires instrumentation that is not common in rural areas. In China, the availability of the non-specific Weill-Felix’s reaction is limited to high-technology settings. However, this method is no longer used as a routine screening method for rickettsial diseases in developing countries. The other drawback to serological assays is that it requires convalescent-phase serum, a requirement that effectively rules out its use for early diagnosis. Molecular diagnostic techniques based on PCR have gradually replaced tedious culture isolation methods for disease diagnosis. Modified PCR techniques, such as nested PCR and real-time PCR, do have their merits; however, these methods are complicated and require a high-precision thermal cycler that makes them impractical for the diagnosis of SFGR infections in rural areas.

In this study, we developed a sensitive LAMP assay that detects a conserved region of the *ompB* gene; the limit of detection of this assay was five copies per reaction or twenty-fold lower than the detection limit for the real-time PCR assay developed in our laboratory (100 copies/reaction) (unpublished data). To evaluate the LAMP assay as a tool for clinical diagnosis, we assayed 11 clinical samples by both LAMP and nested PCR. Of the 11 samples, eight tested positive by the LAMP assay with a limit of detection of five copies per reaction, while none were positive by the nested PCR although the limit of detection was 1 copy/μL. Previous studies have described that the *Bst* polymerase used in LAMP is less sensitive to inhibitors than *Taq* polymerase used in the PCR assay [16-18]. Thus, although nested PCR has been reported to be more sensitive for detecting SFGR than the LAMP assay, we show in this study that the LAMP assay is both more accurate and more sensitive than PCR for screening clinical samples. It was notable; however, that one seropositive sample was not detected by *ompB* LAMP. Because of these observations, we suggest that SFGR infection be confirmed by more than one method to prevent misdiagnosis. Three suspected cases of SFGR were confirmed by LAMP but failed to score as positives by IFA. We suspect that these differences may have resulted from the failure of patients to develop sufficient antibodies for individual differences to be revealed or significant antibody titer during convalescence.

When evaluating the specificity of the *ompB* LAMP assay, we tested 27 members of the order *Rickettsiales* along with several other bacterial pathogens. The results of this study showed that no false-positive amplification was observed with the heterogeneous strains using the SFGR LAMP assay and suggests that LAMP holds promise as an accurate method for differentiating between SFGR and other diseases that cause similar symptoms. The high specificity of LAMP methods has also been reported for other pathogens [16,17,19-21]. Compared with conventional PCR techniques that target between two and four genomic regions, LAMP methods can use between four and six primers to target six to eight regions. Consistent with the work presented here, researchers who study other pathogens have also reported the high specificity of the LAMP assay. The reproducibility of *ompB* LAMP is relatively high, in that both the CVi and CVo are <5%. Although confirming LAMP results by eye is important in low-technology settings and could reduce amplicon carryover leading to false-positive results, this method was less sensitive than agarose gel electrophoresis in our study (data not shown), as well as in others [18]. This result is in contrast to the findings of Mao *et al*. [22], which demonstrated that the two methods yielded consistent results. One concern with the SYBR Green method, however, is the risk of contamination that results from opening the tubes to add reagents. We therefore consider it important to take this risk into consideration when debating how to reduce false-positive results during field testing.

In this study, we detected SFGR DNA in samples extracted from whole blood using the LAMP assay, a kit-based assay that is considered more sensitive than freeze-thaw procedures. The problem of using such a kit, however, is that it would increase the assay cost and turn-around time. In support of using freeze-heat procedures, Paris *et al*. [19] were able to detect *Orientia tsutsugamushi* (order *Rickettsiales*) in DNA samples extracted using either a kit or by freeze-thaw procedures, where 7 out of 7 and 5 out of 7 samples were confirmed by these methods, respectively. Although the kit-based method identified SFGR with a 100% success rate, we feel that the use of boiled samples in the LAMP assay should be reconsidered for greater suitability for use in rural areas.

## Conclusions

A rapid, simple, sensitive, and cost-effective LAMP assay was developed for detecting SFGR in clinical samples. Since the LAMP assay is a fast and sensitive method for testing clinical samples without the aid of sophisticated equipment or complicated operation, this method has the potential to be a powerful tool supporting the diagnosis of SFGR in of China.

## Competing interests

All authors have no competing interests.

## Authors’ contributions

LP carried out the primer design of the LAMP and the performance of the LAMP assay for SFGR, participated in the draft the manuscript. LZ participated in the whole design of the study and performed the statistical analysis and participated in the draft the manuscript. GW collected the cases and sampling and helped to draft the manuscript. QL collected the cases and sampling and helped to draft the manuscript. All authors read and approved the final manuscript.

## Pre-publication history

The pre-publication history for this paper can be accessed here:

http://www.biomedcentral.com/1471-2334/12/254/prepub
